# Exploring the Use of Wearable Sensors and Natural Language Processing Technology to Improve Patient-Clinician Communication: Protocol for a Feasibility Study

**DOI:** 10.2196/37975

**Published:** 2022-05-20

**Authors:** Virginia LeBaron, Mehdi Boukhechba, James Edwards, Tabor Flickinger, David Ling, Laura E Barnes

**Affiliations:** 1 School of Nursing University of Virginia Charlottesville, VA United States; 2 School of Engineering & Applied Science University of Virginia Charlottesville, VA United States; 3 School of Medicine University of Virginia Charlottesville, VA United States

**Keywords:** communication, technology, ubiquitous computing, natural language processing, cancer, palliative care

## Abstract

**Background:**

Effective communication is the bedrock of quality health care, but it continues to be a major problem for patients, family caregivers, health care providers, and organizations. Although progress related to communication skills training for health care providers has been made, clinical practice and research gaps persist, particularly regarding how to best monitor, measure, and evaluate the implementation of communication skills in the actual clinical setting and provide timely feedback about communication effectiveness and quality.

**Objective:**

Our interdisciplinary team of investigators aims to develop, and pilot test, a novel sensing system and associated natural language processing algorithms (CommSense) that can (1) be used on mobile devices, such as smartwatches; (2) reliably capture patient-clinician interactions in a clinical setting; and (3) process these communications to extract key markers of communication effectiveness and quality. The long-term goal of this research is to use CommSense in a variety of health care contexts to provide real-time feedback to end users to improve communication and patient health outcomes.

**Methods:**

This is a 1-year pilot study. During Phase I (Aim 1), we will identify feasible metrics of communication to extract from conversations using CommSense. To achieve this, clinical investigators will conduct a thorough review of the recent health care communication and palliative care literature to develop an evidence-based “ideal and optimal” list of communication metrics. This list will be discussed collaboratively within the study team and consensus will be reached regarding the included items. In Phase II (Aim 2), we will develop the CommSense software by sharing the “ideal and optimal” list of communication metrics with engineering investigators to gauge technical feasibility. CommSense will build upon prior work using an existing Android smartwatch platform (SWear) and will include sensing modules that can collect (1) physiological metrics via embedded sensors to measure markers of stress (eg, heart rate variability), (2) gesture data via embedded accelerometer and gyroscope sensors, and (3) voice and ultimately textual features via the embedded microphone. In Phase III (Aim 3), we will pilot test the ability of CommSense to accurately extract identified communication metrics using simulated clinical scenarios with nurse and physician participants.

**Results:**

Development of the CommSense platform began in November 2021, with participant recruitment expected to begin in summer 2022. We anticipate that preliminary results will be available in fall 2022.

**Conclusions:**

CommSense is poised to make a valuable contribution to communication science, ubiquitous computing technologies, and natural language processing. We are particularly eager to explore the ability of CommSense to support effective virtual and remote health care interactions and reduce disparities related to patient-clinician communication in the context of serious illness.

**International Registered Report Identifier (IRRID):**

PRR1-10.2196/37975

## Introduction

### Background

Effective communication is the bedrock of quality health care, but it continues to be a major problem for patients, family caregivers, health care providers, and organizations [1-4].The ramifications of poor health care communication are profound and can include medical errors [5], suboptimal symptom management [6-9], decreased quality of life for patients and caregivers [10], health care provider distress and burnout [11,12], and inappropriate health care service usage [2]. Effective communication is especially critical in the context of oncology and palliative care, when patients and their families are coping with the stressors of advanced illness and difficult symptoms, such as pain that affects up to 60%-90% of people with cancer [13-15]. Even more problematic is the reality that poor communication related to symptom management contributes to disturbing and unethical health disparities. For example, research has shown that patients from underrepresented racial and ethnic groups are significantly more likely to suffer with undertreated pain [16], die in the intensive care unit when it is not their preference [17,18], and generally experience poorer communication about their health care issues and needs [17,19-22].

Although progress related to communication skills training for health care providers has been made, clinical practice and research gaps persist, including the following: (1) whether the effects are sustained over time [23], (2) which communication training programs are most likely to improve patient care outcomes [23], and (3) the lack of a scalable way to monitor, measure, and evaluate the implementation of communication skills in a natural clinical setting and provide real-time feedback about communication effectiveness [2,24-27]. Leaders in the field, including the Strategic Plan from the National Cancer Institute [28], suggest that to advance the science of communication, we must find ways for continuous, scalable, and clinically meaningful measurement methods [25-27,29,30]. Our protocol helps fill this gap by offering a technology that leverages ubiquitous sensing methods, combined with linguistic and paralinguistic feature engineering methods, to create a novel, scalable, and longitudinal framework to measure the impact of communication in the actual clinical setting tied to a relevant patient outcome such as cancer pain.

Computational methods for processing natural (ie, human) language provide novel opportunities to improve outcomes within health care, including patient-clinician communication. For example, advancements in natural language processing (NLP) technology now make granular analysis of written text and human speech more feasible, allowing us to better parse and understand the dynamics of complex interpersonal interactions [31-34]. This work builds upon prior research regarding NLP analysis of palliative care documentation [31,32,35,36] and evaluation of structured communication skills [37,38], and extends prior work using existing and foundational techniques in NLP and machine learning [39] to implement CommSense. Successful achievement of the aims will establish proof of concept that CommSense can identify relevant verbal (and limited nonverbal) communication signals during patient-clinician interactions, extract relevant metrics of communication performance, and ultimately (long term) provide timely and personalized user feedback to track and evaluate communication performance.

In summary, this proposed research is timely, relevant, and addresses an urgent problem in health care, namely how to assess and measure patient-clinician communication. Improving communication related to cancer pain management can have profound positive implications, such as decreasing patient and family caregiver suffering [40-46], reducing health disparities related to pain management and cancer care [16,19,47-51], improving health care provider job satisfaction [10,52], and mitigating trips to the hospital or emergency department due to uncontrolled pain [53-56]. It is important to emphasize that we recognize the multiple and complex dimensions for improving communication, and many different types of patient-clinician interactions. However, for this initial pilot research, we aim to address 1 aspect, specifically health care provider communication related to cancer pain in the palliative care context, and to determine if we can make a positive impact by building technology to measure and evaluate key features of these types of conversations that can be assessed in real time. Given the scope and intent of this pilot work and the documented need related to this problem, we believe it is an appropriate place to start. If successful, we envision that the CommSense platform will be applicable to a broad range of health care–related conversations and contexts.

### Preliminary Work

CommSense will build upon an existing Android smartwatch platform (SWear) developed by coauthors (LB and MB) to collect sensor signals from smartwatches [57]. Prior work using SWear has demonstrated acceptance of the technology, accuracy of the underlying NLP technology, and the ability to successfully use the platform across multiple contexts and study samples [58-64]. Specifically, the SWear platform has been previously used to evaluate communication in socially anxious individuals, and the feasibility and ability of Swear to extract audio features (eg, energy, pitch, Mel-frequency cepstral coefficients and NLP features such as sentence representation from pretrained Roberta/Sentence Transformers, term frequency-inverse document frequency) for predicting different anxious states during natural conversations have been established. Although this study addresses a different clinical problem, the foundational and established NLP methods, and the techniques for collecting, storing, and processing audio data are similar. This research leverages our team’s complementary skillsets related to smartphone-based biomarkers of cognitive states and virtual human training systems for patients, clinicians, and teachers (engineering, LB [65-69]); mobile and ubiquitous computing, NLP technology, machine learning (engineering, MB [66]); patient-provider communication and smart health (nursing, VL [70-73]; medicine, TF [74-77]); informatics (medicine, DL [78,79]); and oncology and pain management (nursing, VL [73,80-82]). Our team possesses the clinical and technical expertise necessary to support the aims of this research.

### Aims

This is a 1-year (November 2021-November 2022) “proof-of-concept” feasibility study to develop a novel ubiquitous system and associated algorithms for measuring quality of conversations (CommSense) that can (1) be implemented on mobile devices, such as smartwatches; (2) reliably capture patient-clinician interactions in a clinical setting; (3) process communication to extract key markers of communication effectiveness and performance; and (4) ultimately (long term) provide real-time feedback to end users to improve communication and patient health outcomes.

## Methods

### Specific Aim 1: Establish Feasible Metrics of Communication to Extract From Conversations (Months 1-3) 

#### Data Collection

Clinical investigators will conduct a thorough review of the recent health care communication and palliative care literature to develop an evidence-based “ideal and optimal” list of communication metrics. We anticipate that this list will have two categories: (1) general best practices of health care communication (eg, verbal metrics such as the amounts of silence or pauses; speaking turns, interruptions, and overtalking; open-ended versus closed-ended questions; and nonverbal metrics, such as eye contact; arms crossed or open; sitting or standing) and (2) metrics more specific to conversations about cancer pain management (eg, assessment questions related to the severity, onset, and quality of the pain). We will organize this list conceptually around the 6 recommended domains to operationalize patient-centered palliative cancer care communication as detailed by McCormack et al [25,83], including exchanging information, fostering healing relationships, recognizing and responding to emotions, managing uncertainty, making decisions, and enabling patient self-management. We will also organize this list by “need,” “nice,” and “next” to record CommSense features considered essential by the clinical team, including the preferred and future features. This initial list of communication metrics will encompass the “what” (content questions) and the “how” (in what manner the questions are asked). This list will be discussed collaboratively within the study team and consensus about the included items will be reached. The list will also be vetted with other communication experts in the field, with whom investigators of this project have established relationships.

### Specific Aim 2: Develop the CommSense Software (Months 4-7)

#### Data Collection

**Figure 1:**
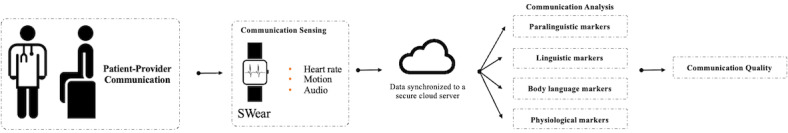
CommSense system overview. Data are captured during patient-clinician interactions using smartwatches and synchronized to the secure cloud server to extract metrics characterizing communication quality, such as linguistic and paralinguistic markers (primary focus of the study) and physiological markers (secondary focus of study).

The “ideal and optimal” list of communication metrics will be shared with engineering investigators to gauge technical feasibility. We anticipate this will be a highly iterative process between the engineering and clinical team investigators to refine our list of desired communication metrics based upon technical capabilities and clinical relevance. As discussed above (see Preliminary Work), CommSense will build upon prior work using an existing Android smartwatch platform (SWear) to collect sensor signals from smartwatches [57]. SWear collects multiple sensor streams such as motion, audio, and physiological data and synchronizes the data to a secure server for further analysis. SWear can also deliver microsurveys (Ecological Momentary Assessments) for collecting self-reported data. SWear has already been validated in multiple studies and is available on the Android play store [57,66]. CommSense will include sensing modules that can collect (1) physiological data via built-in sensors to measure variables such as heart rate, (2) gesture data via accelerometers and gyroscope sensors, and (3) voice data via the embedded microphone (see Figure 1). Although we recognize the importance of nonverbal communication and will thus leverage the existing passive sensing capabilities of CommSense to collect data related to heart rate variability and movement, these markers will be secondary to our primary focus of collecting audio data to analyze verbal and linguistic metrics of patient-provider communication.

### Specific Aim 3: Pilot Testing the Ability of CommSense to Accurately Extract Identified Communication Metrics (Months 8- 12)

#### Data Collection

CommSense will be piloted with 5 nursing or medical students and 5 experienced oncology/palliative clinicians (n=10) using simulated scenarios to evaluate its accuracy in capturing and extracting the preidentified communication metrics (Table 1). Each participant will work through 2 conversation scenarios (n=20,10 per group [84,85]) and we will collect multiple data points related to paralinguistic and linguistic markers, as well as body language and physiological markers (Figure 1). The primary goal of Aim 3 is to verify the fidelity of the data captured using CommSense by comparing findings to ground truth. Clinical team members will write 2 relevant scripted scenarios (approximately 10-15 minutes in length) that relate to assessing and managing cancer pain in a palliative care context. It is critical to emphasize that although we ultimately aim to advance communication evaluation beyond scripted and simulated scenarios, this pilot study represents the foundational first step to develop technology that can reliably capture and analyze communication data before being implemented “in the wild.” Consistent with the scope of an exploratory pilot, this initial research will not involve real patients with protected health information. Future work with CommSense that involves actual patients will address all relevant privacy measures and the regulations of the Health Insurance Portability and Accountability Act of 1996. Pilot testing will occur in the institution’s Clinical Simulation Labs. After consent and basic demographic data are obtained, participants will wear CommSense and enact the 2 scenarios. The “patient” for our pilot testing may be a voice-capable mannequin, a member of our study team, or an experienced clinician volunteer, depending on what is feasible considering COVID-19 restrictions. The interaction will be recorded by CommSense and by a separate fixed external microphone and recording device to establish ground truth. At the end of the interaction, participants will complete a brief survey to assess the acceptability of using CommSense, provide suggestions for future iterations, state preferences regarding data sharing, and rate their self-perceived communication performance.

**Table 1:** Examples of anticipated features to extract and analyze from conversations using CommSense.

Feature		Communication goal or rationale
**Audio signal variables**		
	Silence	To allow time to process complex or difficult information
	Speaking turns and interruptions	To avoid speech dominance and ensure all participants are heard
	Prosody, flow, and rhythm	To reduce stress, and increase empathy and clarity
**Natural language variables (primary)**		
	Complexity of language	To avoid medical jargon to decrease confusion
	Tone or sentiment	To convey empathy, warmth, and openness, and build rapport
	Open-ended versus close-ended questions	To allow exploration and promote bidirectional dialogue
	Language associated with communication best practices related to palliative care and pain management (eg, “I want to be sure I understand…;” “It sounds like you are feeling…;” “Can you tell me more about…”)	To use language associated with therapeutic communication related to symptom management in the context of serious illness
**Nonverbal variables^a^ (secondary)**		
	Heart rate, motion or movement, and gestures	To use nonverbal indicators (such as sitting down and not crossing arms) for establishing rapport, trust, and dialogue between the patient and provider, and heart rate for indicating provider stress level during conversation

^a^Due to the capability of the sensing platform and ease of collecting the data, nonverbal physiological and gesture-related variables will be collected, but they are not the primary focus of this study.

#### Data Analysis

CommSense data will be first preprocessed to clean the sensor data and extract markers of communication quality based on the metrics established in Aim 1. This will be achieved by analyzing (1) paralinguistic markers from the audio signals such as pitch, energy, and Mel-frequency cepstral coefficients to characterize features such as tone, silence, and speaking turns [86,87]; and (2) linguistic markers from the audio signals by first parsing the signal to text using Google’s speech-to-text application programming interface. Then, several semantic features will be extracted using NLP methods such as word embedding features that can describe structural organization of words in the conversation (eg, frequency-based methods such as count vector and term frequency-inverse document frequency) and lexical features such as linguistic inquiry and word count, which is one of the most popular lexical feature extraction methods that has been rigorously validated in the context of psychometric analysis of textual data. We will also explore passively collected motion and physiological data, such as motion and heart rate data, and extract features that can characterize stress, such as heart rate variability. Finally, all the extracted linguistic, physiological, and motion-derived features will be analyzed to study how they fluctuate across scenarios and groups using a multilevel analysis given the hierarchical structure of the data.

#### Establishing Ground Truth

Externally recorded conversations will be transcribed, verified, and then compared to the CommSense-generated output for the same conversation. To conduct this comparison, we will proceed in a stepwise manner. First, hard copy transcripts of the audio-recorded conversation will be independently coded using qualitative software by 2 clinical investigators to identify the communication metrics that we expect to be extracted by CommSense. Second, the results obtained by the 2 investigators will be compared to establish interrater reliability. If there is a discrepancy, a third team member will be consulted. Given concerns regarding the use of the Cohen κ [88] to evaluate interrater reliability, we chose verbal discussion to reconcile any potential disagreements between investigators regarding communication metrics identified in the transcripts. Third, we will compare the generated CommSense output with the ground truth investigator review of the same conversation. We will do this by assigning a numerical value based on the concordance between the CommSense output and the investigator review of the transcript. For each identified communication metric (eg, instances of overtalking) we will assign a score of 0 if the CommSense output does not match the investigator review of the hard copy transcript and a score of 1 if it does. For example, with 10 identified metrics, the best possible concordance score would be 10, meaning the CommSense output and the hard copy transcript review achieve 100% concordance. We will then calculate concordance scores for each conversation metric to achieve a composite score for each conversation to explore how accurately the CommSense software is able to extract the desired metrics from palliative care conversations. Descriptive statistics will be used to summarize demographic data and participant responses to the postdeployment survey assessing the acceptability of CommSense.

### Ethics Approval

Institutional Review Board (University of Virginia Social & Behavioral Sciences IRB, #4985) approval has been granted.

## Results

Work began on Specific Aim 1 in November 2021 and Specific Aim 2 in April 2022. Participant recruitment is expected to begin in summer 2022. We expect preliminary results to be available in fall 2022.

## Discussion

### Potential Applications of CommSense

We hypothesize that it will be feasible to extract relevant metrics of communication performance with >80% concordance between ground truth audio transcripts and the CommSense-generated output. We also hypothesize that health care providers will consider CommSense acceptable and helpful in improving their communication skills.

This pioneering idea represents a paradigm shift in health care delivery by leveraging a scalable and novel technology to measure, track, and evaluate patient-provider communication in the clinical setting. Consider the following potential scenario: A team of oncology health care providers from Clinic A complete intensive communication skills training and learn structured techniques to improve their ability to have difficult conversations with patients coping with advanced cancer. During the training, they practice these skills using scripted role plays. After the training, the health care providers in Clinic A are each given a CommSense wristband sensor (eg, smartwatch) to wear as they care for patients with advanced cancer in the actual clinic setting. With consent from all participants, the wristband sensor, equipped with multimodal sensors to capture natural language, is activated during patient-clinician conversations, and on-board processors capture, extract, and summarize preprogrammed metrics of interaction quality. Following the clinic visit, the wrist-worn device uploads summarized communication performance metrics for real-time display to the physician, nurse, and social worker. The physician is shocked to see that her speaking turns occupied 89% of the visit time and that she interrupted the patient 15 times. The nurse is pleased to see that her use of medical jargon has decreased to a single instance; however, she asked 3 times as many closed-ended questions compared to open-ended questions. The social worker realizes that yet again, he was the only health care provider who engaged the caregiver in discussing the pain management plan or addressed safe opioid handling practices at home. The entire team can collectively observe that their communication performance was not as strong as last week, but they are still performing at a higher level than 75% of their peer clinics. Additionally, as the individual and team communication performance in Clinic A improves, patients report lower pain scores. The trainers who led the communication skills training can track the progress of participants over time, assess the retention of communication skills over time, see how skills are being implemented “in the wild,” and based on the results, they can better tailor future training sessions to meet the needs of participants. The hospital administrator is thrilled when she receives a monthly report that clearly shows oncology clinics using CommSense sensors have higher patient and caregiver satisfaction scores, fewer medication errors, and lower readmission rates.

The above scenario paints the long-range vision and potential impact of the CommSense technology. Although we propose to begin with 1 aspect of cancer care communication (pain management), we believe this model will be generalizable to other health care settings and contexts where quality communication is essential (eg, other types of high-stakes interactions, such as goals-of-care conversations or death notifications). We are eager to test CommSense in populations at high risk for health disparities and communication barriers (eg, patients from underrepresented groups experiencing undertreatment of pain), English as second language patients, and those at high risk for distressing symptoms (eg, patients with metastatic cancer). We also see key opportunities to customize this intervention for different metrics depending on the communication context, relevant outcome measures and communication preferences, and the needs and goals of participants. For example, different communication strategies may be needed for a patient who is an artist with pancreatic cancer, does not speak English, and is being treated in a rural community hospital, compared to the communication strategies needed for a patient who is a physician, speaks English fluently, and is being treated at an academic medical center for routine gallbladder surgery.

### Dissemination Plan

Results from this research will be presented at relevant technical and clinical academic conferences, as well as published in scholarly peer-reviewed journals. As we are a highly interdisciplinary team, our dissemination plan will aim to reach diverse audiences within nursing, medicine, and engineering domains. We also anticipate sharing findings with other key stakeholders (eg, clinicians, hospital administrators, and cancer advocacy groups) in more informal settings to continue developing the CommSense platform.

### Limitations

The primary limitation of this study is that it is being conducted in simulated clinical scenarios rather than in real clinical settings. Although this is an important limitation to acknowledge, it is appropriate and essential to validate the functionality and feasibility of CommSense in simulated settings before implementing it in an actual patient care setting. Another important limitation is the inability of CommSense to completely capture the important and complex nuances (eg, subtle nonverbal cues) of patient-clinician communication.

### Conclusions

This pilot study is poised to make a valuable contribution to communication science, and NLP and wearable computing capabilities. We see particularly exciting options to apply the CommSense technology to support effective virtual and remote patient-clinician interactions, which are likely to become more normative in our post-COVID world. Future steps include leveraging results to (1) implement CommSense in real clinical scenarios with a larger sample of participants to more robustly assess its feasibility and acceptability; (2) test CommSense with diverse high-risk, high-need populations and contexts and explore how to best customize the system using communication metrics tailored to patient, clinician, and organizational needs; (3) evaluate the impact of CommSense on relevant patient-centered and organizational outcomes, such as patient pain, medical errors, staff turnover, goal-concordant care and health care usage; (4) conduct experimental trials to test the effectiveness of CommSense in terms of pre- and postcommunication training versus standard of care; (5) determine how to best capture, integrate, and display nonverbal data relevant to communication such as position, eye contact, and gestures; (6) test multiple concurrent users of CommSense (eg, the clinician, patient, and family caregiver wearing CommSense during a conversation and receiving feedback) to evaluate the interactional aspects of communication; and (7) identify how to most effectively share collected data with key stakeholders (patients, caregivers, health care providers, and organizational units).

## Appendix

Multimedia Appendix 1 Peer-review report by The Center for Engineering in Medicine, University of Virginia - Engineering-in-Medicine Seed Grant Program (Virginia, USA).

## Abbreviations

NLP: natural language processing
