# Supplementary material for: Exploring the Use of Wearable Sensors and Natural Language Processing Technology to Improve Patient-Clinician Communication: Protocol for a Feasibility Study
Source: JMIR Res Protoc. 2022 May 20;11(5):e37975. doi: 10.2196/37975 (PMC9166632; doi:10.2196/37975)
Supplement: Multimedia Appendix 1 [file resprot_v11i5e37975_app1.pdf]

ENGINEERING-IN-MEDICINE SEED GRANT PROGRAM
Review Summary

Project Title

Exploring the Use of Wearable Sensors and Natural Language Processing Technology to Improve Provider-Patient Communication

Co-PIs

LeBaron (SON); Flickinger (SOM – GGPH); Boukhechba (SEAS – ESE)

Ratings:

Fostering Collaboration:	**Reviewer 1:** Top 10%	**Reviewer 2:** Top 25%
Lead to External Funding:	**Reviewer 1:** Top 25%	**Reviewer 2:** Top 25%
Scientific/Clinical Impact:	**Reviewer 1:** Top 25%	**Reviewer 2:** Top 50%
Overall:	**Reviewer 1:** Redevelop	**Reviewer 2:** Redevelop

EIM Feedback to co-PIs

In the current round, we received 22 applications and funded 7. The next few proposals – including this one – were considered good candidates for further development and potential resubmission in a future round. We have provided reviewer comments below, and would be happy to meet with you to discuss and provide further guidance on a resubmission.

**Please note that Fall 2021 currently stands as the last round of seed grants for EIM. We will only be accepting proposals from those teams from Spring 2021 and have been recommended for 'Redevelop.'

Reviewer Feedback to co-PIs

Reviewer 1

Overall, I think the outcome of this project (e.g., the CommSense) will generate some impacts on clinical practice. However, the scientific contribution of this proposal is not convincing. For example

- In specific aim 1, it is not clear how to implement these communication metrics in practice. If these metrics are implemented based on some existing NLP systems/methods, which are the accuracies of these systems/methods?

- In specific aim 3, it is unclear the scale of the data collection. For data-driven approaches, ten data points seem to be far away from enough. Or, maybe there are some other strategies to collect more data that I somehow missed from this proposal?

Reviewer 2

Very interesting proposal with good use of existing software to expand use in the health care setting.

Questions:

1. Will these biosensors pick up changes in providers (medical / nursing) during these conversations. My previous experience in this realm is that medical and nursing students were for the most part healthy and did not produce changes in the biosensors that were meaningful.

2. The CommSense application has been validated in the SWear format with socially anxious individuals. Will this be able to extrapolate to the medical professionals you hope to study?

3. Do you think patients will allow recording of their conversations for studying and examination when you are beyond the pilot program?

The enthusiasm from the NSF and NIH program directors is exciting.

REVIEWER 1: Critique: Unclear how communication metrics will be implemented in clinical practice; if these metrics are based on existing NLP systems/methods more detail is needed regarding the accuracy of such systems/methods. **Response:** The *CommSense* application will not need to be built from scratch, but instead will involve designing new software and associated algorithms on top of an existing Android smart watch platform (SWear) developed and tested by co-investigators Barnes and Boukhechba (SEAS). Prior work with SWear has demonstrated *acceptance* of the technology, *accuracy* of the underlying NLP technology, and the ability to successfully use the platform *across multiple contexts* and study samples. Additional details have been added to the 'Preliminary Work' section under 'Project Team.' It is also important to note that although *CommSense* builds upon known and validated NLP systems and methods, it is a key goal of this pilot to explore how accurately CommSense can identify quality communication metrics that can be used to evaluate and assess real-time patient-provider interactions. Please see the "Impact" section of the proposal for a detailed example of how *CommSense* communication metrics could be implemented in clinical practice.

Critique: Scale of data collection is unclear. Concern related to participant sample size. **Response:** 10 participants is an appropriate sample size for a 1-year pilot study in which the goal is to establish proof of concept (versus testing for statistical significance)[1,2]. It is also important to emphasize that each participant will generate multiple and numerous data points. For example, each participant will pilot test *CommSense* with 2 scripted conversations, therefore 10 participants will generate 20 conversations. For each conversation (n=20), we will gather multiple data points regarding paralinguistic and linguistic markers, as well as body language and physiological markers (see Figure 1 and clarifying details added to Specific Aim 3, Data Collection). Lastly, establishing Ground Truth (Aim 3) for this study will be a fairly labor-intensive process and a sample size of 10 participants (and 20 conversations) is realistic given the scale, scope, and budget of this pilot project.

REVIEWER 2: Critique: Concern whether biosensors will detect meaningful changes in likely healthy nursing and medical student participants. **Response:** While we do have the capability and plan to passively collect basic physiological data using the commercial Android smartwatch on which *CommSense* is deployed, it is important to re-iterate that physiological/non-verbal data are not the primary focus of our study. This has been clarified within Specific Aim 2, Data Collection; Figure 1 and Table 1. Instead, the primary focus for our pilot study is to accurately collect and analyze linguistic markers that can be utilized to evaluate the quality of patient-provider conversations. Any non-verbal data gathered through biosensors will not be used to extrapolate information related to the participant's overall health but could provide preliminary insights regarding non-verbal aspects of communication that could help inform next steps, contextualize our results, or inform additional questions, such as, do physiological data differ between experts/novices, such as senior versus junior faculty or between student versus experienced clinicians.

Critique: The CommSense application has been validated in the SWear format with socially anxious individuals. Will this be able to be extrapolated to the medical professionals you hope to study? **Response:** The SWear platform and associated algorithms provide the foundation from which we will build the *CommSense* application. SWear has been validated in multiple studies, including measuring wellbeing for elderly, analyzing social anxiety from audio data, analyzing the effect of route context on driving behaviors, and measuring fluid consumption of kidney patients, and many others. A key advantage of the SWear platform is that its core functionality can be applied across multiple contexts and samples – e.g., socially anxious individuals or individuals engaging in medically related conversations. We have added clarifying information and references within the section 'Preliminary Work' that describes prior feasibility and acceptability of the SWear platform and how it can be applied in this study population.

Critique: Do you think patients will allow recording of their conversations for studying and examination when you are beyond the pilot program? **Response:** Based on prior related research that has involved audio-recording of highly sensitive patient-provider conversations (such as Lee Ellington's work recording conversations between hospice patients/families and hospice care nurses[3–5] or James Tulsky's work recording conversations between providers and patients with serious illness[6,7]), as well as a large body of literature that demonstrates both patients' and providers' desire for improved communication (see Background and Significance), we hypothesize *CommSense* will be acceptable beyond the pilot program. To help validate this, at the end of the interaction participants will complete a brief Qualtrics survey to assess acceptability of using *CommSense*, suggestions for future iterations, preferences regarding data sharing, and to rate their self-perceived communication performance (see Specific Aim 3, Data Collection).

[1]Hertzog MA. Considerations in determining sample size for pilot studies. *Res Nurs Health*. 2008; [2]Julious SA. Sample size for a pilot study. *Pharm Stat*. 2005; [3]Ellington et al. Communication among cancer patients, caregivers, and hospice nurses. *Patient Educ Couns*. 2018; [4]Clayton MF et al. Communication behaviors and patient and caregiver

emotional concerns. *Oncol Nurs Forum*. 2014; [5]Reblin et al. In-home conversations of couples with advanced cancer *Psychooncology*. 2020; [6]Anderson WG…Tulsky JA. Code status discussions. *J Gen Intern Med*. 2011; [7]Pollak KI… Tulsky JA, et al. Physician empathy and listening. *J Am Board Fam Med*. 2011.

ENGINEERING-IN-MEDICINE SEED GRANT PROGRAM
Review Summary

Project Title

Exploring the Use of Wearable Sensors and Natural Language Processing Technology to Improve Provider-Patient Communication

Co-PIs

LeBaron (SON); Barnes (ESE – SEAS); Boukhechba (ESE – SEAS); Flickinger (GGPH – SOM); Ling (GGPH-SOM)

Ratings:

Fostering Collaboration:	**Reviewer 1:** Top 10%	**Reviewer 2:** Top 10%
Lead to External Funding:	**Reviewer 1:** Top 10%	**Reviewer 2:** Top 10%
Scientific/Clinical Impact:	**Reviewer 1:** Top 10%	**Reviewer 2:** Top 25%
Overall:	**Reviewer 1:** Fund	**Reviewer 2:** Fund

EIM Feedback to co-PIs

This effort will bring together investigators from SON (LeBaron), SOM (Flickinger, Ling) and SEAS (Barnes, Boukhechba). We agree with reviewer comments that you have clarified project aims and have strong plans for student experiences as well as plans for federal follow-on funding.

Reviewer Feedback to co-PIs

Reviewer 1

Overall, I think this is a very strong proposal regarding its scientific novelty and societal impacts. I don't have any major concern about the proposed idea, and only two minor comments on some technical details:

- Page 5: the term "frequency-based embedding methods" is not accurate, neither Count Vector nor TF-IDF belongs to embedding-based methods.
- In the same paragraph, the idea of "few-shot learning" is intriguing. However, as there is no explicit description of a machine learning component anywhere else in the proposal (regarding what is the machine learning problem and what are its inputs/outputs), it is unclear to me why few-shot learning is necessary and in which component it will be used.

Reviewer 2

Appreciate the extra explanation about the results that are gathered and the use of the program. As this is just a pilot program, I am not sure it will have a significant impact, but it is a good step towards determining if this program and NLP will be useful in improving communication. I do think there is potential for outside funding, as patient centered research is quite important in our field.
